# Genome-wide association study for grain yield and related traits in elite wheat varieties and advanced lines using SNP markers

**DOI:** 10.1371/journal.pone.0188662

**Published:** 2017-11-27

**Authors:** Sheng-Xing Wang, Yu-Lei Zhu, De-Xin Zhang, Hui Shao, Peng Liu, Jian-Bang Hu, Heng Zhang, Hai-Ping Zhang, Cheng Chang, Jie Lu, Xian-Chun Xia, Gen-Lou Sun, Chuan-Xi Ma

**Affiliations:** 1 College of Agronomy, Anhui Agricultural University; Key Laboratory of Wheat Biology and Genetic Improvement in the Southern Yellow & Huai River Valley, Ministry of Agriculture, Hefei, Anhui, China; 2 Institute of Crop Science, National Wheat Improvement Centre/The National Key Facility for Crop Gene Resources and Genetic Improvement, Chinese Academy of Agricultural Sciences (CAAS), Beijing, China; 3 Department of Biology, Saint Mary’s University, Halifax, Nova Scotia, Canada; Institute of Genetics and Developmental Biology Chinese Academy of Sciences, CHINA

## Abstract

Genetic improvement of grain yield is always an important objective in wheat breeding. Here, a genome-wide association study was conducted to parse the complex genetic composition of yield-related traits of 105 elite wheat varieties (lines) using the Wheat 90K Illumina iSelect SNP array. Nine yield-related traits, including maximum number of shoots per square meter (MSN), effective number of spikes per square meter (ESN), percentage of effective spike (PES), number of kernels per spike (KPS), thousand-kernel weight (TKW), the ratio of kernel length/kernel width (RLW), leaf-area index (LAI), plant height (PH), and grain yield (GY), were evaluated across four environments. Twenty four highly significant marker-trait associations (MTAs) (*P* < 0.001) were identified for nine yield-related traits on chromosomes 1A, 1D, 2A (2), 3B, 4A (2), 4B, 5A (4), 5B (4), 5D, 6B (2), 7A (2), and 7B (3), explaining 10.86–20.27% of the phenotypic variations. Of these, four major loci were identified in more than three environments, including one locus for RLW (6B), one locus for TKW (7A), and two loci for PH (7B). A cleaved amplified polymorphic sequence (CAPS) marker *Td99211* for TKW on chromosome 5A was developed and validated in both a natural population composed of 372 wheat varieties (lines) and a RIL population derived from the cross of Yangxiaomai × Zhongyou 9507. The CAPS marker developed can be directly used for marker-assisted selection in wheat breeding, and the major MTAs identified can provide useful information for fine-mapping of the target genes in future studies.

## Introduction

Wheat (*Triticum aestivum* L.) is one of the most important and widely-grown staple crops. The continuous decrease in farmland and rapid increase in population results in big problems regarding the production of sufficient food to meet the global demand. A previous study examining food security suggested that food production would need to increase by 70–100% in 2050 [1]. Wheat producers and breeders have managed a considerable increase of yields over the last 50 years, reaching more than 700 Mt in 2016. Nevertheless, increasing grain yields is still a primary objective in wheat breeding [2]. The complex genetic relationships between yield and related traits (e.g., plant height, number of spikes per hectare, number of kernels per spike, and thousand-kernel weight) need to be clarified to achieve further breakthroughs to develop high-yielding wheat varieties.

Wheat yield and related traits are controlled by multiple quantitative trait loci (QTLs), and vulnerable to environmental factors. To date, a considerable number of QTLs associated with grain yield and related traits have been detected on almost all 21 wheat chromosomes [3–10]. On the other hand, many marker-trait associations (MTAs) for grain yield and related traits have also been identified under various genetic backgrounds [11–20]. Compared with bi-parental population mapping largely limited by bi-parental genetic background, genome-wide association study (GWAS) based on linkage disequilibrium is an effective approach to identify abundant genetic loci for complex traits in diverse natural populations because of their abundant genetic backgrounds [20–21].

The available genotyping tools have rapidly developed for QTL mapping and GWAS, from wheat simple sequence repeat (SSR) markers to diversity array technology, then to 9K, 90K, 660K and 820K SNP arrays. The high-density SNP arrays have been widely used to identify MTAs in GWAS for grain yield and related traits, and most MTAs for these traits have been identified on chromosomes 1A, 1B, 2B, 3A, 3B, 4A, 5A, 5B, 6A, 7A, and 7B [11–20]. These MTAs provided useful information for identification of grain yield genes in wheat.

In our previous research, genetic diversities of 190 wheat varieties and advanced lines were characterized using SSR markers; these varieties were selected from three major wheat-growing regions in China, i.e., Yellow & Huai Rivers Valley, Middle and Lower reaches of the Yangtze River, and southwestern China [22]. Among these, we further selected 105 representatives with large genetic diversity and wide use in breeding program to identify major MTAs associated with grain yield and related traits via GWAS using wheat 90K Illumina iSelect SNP array. A cleaved amplified polymorphic sequence (CAPS) marker for thousand-kernel weight (TKW) was further validated in 372 wheat varieties (lines) and 188 lines from Yangxiaomai × Zhongyou 9507 RIL population. The results will be useful for improvement of grain yield in wheat breeding.

## Materials and methods

### Plant materials and field trials

The association mapping panel comprised 105 elite wheat varieties and advanced lines with abundant phenotypic variations (S1 Fig) from three major winter wheat -growing regions in China, i.e., Yellow & Huai Rivers Valley, Middle and Lower reaches of the Yangtze River, and southwestern China, based on genetic diversities [22]. A natural population composed of 372 wheat varieties (lines) and a RIL population (188 lines) derived from the cross of Yangxiaomai × Zhongyou 9507 were used to validate the association of a CAPS marker (*Td99211*) with TKW on chromosome 5A.

The association mapping panel was planted at the Dayangdian experimental farm of Anhui Agricultural University in Hefei (31°93′N, 117°21′E) and the Guohe experimental farm of Anhui Agricultural University in Lujiang (31°47′N, 117°25′E) during the 2014–2015 (designated as E1 and E2, respectively) and 2015–2016 (designated as E3 and E4, respectively) cropping seasons. Each plot comprised five 4.0-m rows spaced 25 cm apart. The Natural population and the RILs population were planted at the Dayangdian experimental farm of Anhui Agricultural University in Hefei (31°93′N, 117°21′E) with two 2.0-m rows spaced 25cm apart per material during the 2015–2016 and 2016–2017 cropping seasons. Field trials were conducted in randomized complete blocks with two replications. Test plots were managed according to local practices. All fields were kept free of diseases and weeds.

### Phenotypic trait evaluation and statistical analysis

The 1,300 seeds were sown in each plot. At the two- or three-leaf stages, seedlings were counted and thinned for about 1,000 evenly distributing plants each plot. Three 1.0-m sections were chosen and marked in each plot. The maximum number of shoots per section was scored during the elongation stage, and then converted to maximum number of shoots per square meter. The leaf-area index (LAI) was measured with five replications per plot at the heading stage using the SunScan Canopy Analysis System (Delta-T Devices Ltd., Burwell, Cambridge, UK). The effective number of spikes per square meter (ESN) was counted at the ripening stage, and then the percentage of effective spikes (PES) was calculated. Plant height (PH) was measured with five replications per plot at the yellow maturity stage, and the mean value of five scores was used for subsequent analysis.

When plants reached physiological maturity, 20 spikes per plot were harvested and manually threshed. The total number of kernels was determined using the WSeen SC-G Seed Test System (WSeen Testing Technology Co., Ltd., Hangzhou, China), and then converted to number of kernels per spike (KPS). The remaining spikes in each plot were harvested and threshed using Wintersteiger Plot Combines (Wintersteiger AG, Ried i.I., Austria). Grain yield (GY) was calculated as the weight of wheat grain harvested from whole plots. TKW was measured using 1,000 randomly selected kernels with two replicates, while the ratio of kernel length/kernel width (RLW) was determined using 100 correctly placed kernels in the WSeen SC-G Seed Test System in duplicate.

### Statistical analysis of phenotypic data

The best linear unbiased predictions (BLUPs) can eliminate the environmental deviation and estimate the real individual breeding value, so it has gradually become more common application by plant breeders who wish to generate more precise estimates of genotypic values [23–25]. Therefore, the broad sense heritability (*H*_*B*_^*2*^) and BLUPs were determined using the ‘lme4’ package of the R3.1.3 software (www.r-project.org), with year and location as random effects in the model [Y = lmer (X~(1|LINE) + (1|LOC) + (1|YEAR) + (1|LINE:LOC) + (1|LINE:YEAR)] [26–27]. The descriptive statistics of different traits, correlations between BLUP and the measured values among different environments, and those among BLUPs of different phenotypes were analyzed using SPSS Statistics 20 (http://www.ibm.com/analytics/).

### DNA extraction and genotyping

Dry seeds were ground to powder using the FastPrep-96^™^ Homogenizer (MP Biomedicals, USA) for genomic DNA extraction following the MPure Nucleic Acid Purification system (MP Biomedicals). The DNA quality was checked by 1.0% agarose gel electrophoresis, and the concentration was determined with the NanoDrop ND-200 Nanophotometer (Thermo Fisher Scientific Inc., USA).

Samples were genotyped using the wheat 90K Illumina iSelect array containing 81,587 SNP markers at Beijing Compass Biotechnology Co., Ltd., following the manufacturer’s protocol [28], in which 46,977 SNPs were genetically mapped using eight mapping populations [29]. The SNP allele clustering and genotype calling were completed with the Genome Studio program (version 2011.1) (Illumina; https://www.illumina.com/). The accuracy of the SNP clustering was visually validated, and incorrectly clustered SNPs were manually adjusted. The SNP markers with a missing rate exceeding 0.1 or a minor allele frequency less than 0.05 were removed, and 31,250 effective SNPs were used for subsequent population structure, principal component, and kinship analyses. Of these, 15,430 SNP markers mapped on different chromosome regions based on the genetic and physical maps [29] (S1 Table) were used for further genome-wide association analysis.

### Population structure, principal component, and kinship analyses

The population structure of the association mapping panel was assessed with all 31,250 effective SNP markers on 21 wheat chromosomes, using the fastStructure algorithm in Python (http://rajanil.github.io/fastStructure/). Multiple *K* values ranging from 1 to 10 were implemented using the Simple Model prior to obtaining a reasonable range of values for the appropriate model complexity required to explain the population structure [30]. A useful heuristic technique based on the tendency of mean field variation schemes was used to select *K* [30]. The estimated *Q* matrix was obtained based on a variation inference executed for a choice of *K*, and the ancestry contribution of each model component was computed as the mean admixture proportion for all samples [30].

The principal component analysis (PCA) was conducted with numerical values for genotypes (31,250 SNP markers) using the genome association and prediction integrated tool (GAPIT) of the R software [31–32]. A turning point of the eigenvalue change was chosen as the optimal number for the principal component (PC).

The marker-based kinship matrix (K*) was calculated with the same genotypes using the VanRaden method, and then used to create a clustering heat map of the association mapping panel in the GAPIT [32].

### Genome-wide association analysis

A GWAS was performed using the mixed linear models in TASSEL (version 5.2) [33–34]. The significant MTAs between 15,430 SNPs and traits were identified using a model with a *Q* matrix as the fixed effect and a kinship matrix as the random effect (*Q* + K*) [35] as well as a model with a PC matrix as the fixed effect and a kinship matrix as the random effect (PCA + K*), at a threshold of *P* < 0.001. Because the degree of the correlation with different models varied from trait to trait, a Bayesian information criterion (BIC)-based model comparison was used for each phenotype [36]. The criterion value for a model was calculated as BIC = −2·maximized log-likelihood + log(*n*)·number of estimated parameters (*n* = sample size). The model with *Q* + K* or PCA + K* as covariates was selected for each trait according to the maximum BIC value [37–38]. The SNPs with the genetic distance less than 5 cM were assumed as one MTA, and the MTA identified in more than three environments (*P* < 0.001) was assumed as a major locus.

Based on a SNP (*Tdurum_contig71499_211*) on chromosome 5A significantly associated with TKW (*P* < 0.001), a CAPS marker was developed, designated *Td99211* (F: GCTGGAGCAAAGTTGTATT, R: GGTTATGTCGCTTGAGTTAT), using Primer premier 5.0. PCR was performed in a total volume of 10 μL, including 1.0 μL of 10 × PCR buffer, 200 μM of dNTPs, 4 pmol of each primer, 0.5 U *Taq* DNA polymerase and 100 ng of template DNA. The PCR procedure included a denaturation at 94°C for 5 min, followed by 38 cycles of denaturation at 94°C for 30 s, 60°C for 30 s, 72 `C for 30 s, and a final extension at 72°C for 8 min. The PCR products were digested with *AluI* at 37°C for 3 h (restriction site: AG/CT, http://www.neb-china.com) according to the manufacturer’s directions, and separated on 1.5% agarose gel. The SPSS Statistics 20 software was used for data analysis, and *t*-tests were performed using the independent-samples *t*-test.

## Results

### Grain yield and related traits

The phenotype values of GY and related traits (i.e., MSN, ESN, PES, KPS, TKW, RLW, LAI, and PH) of the association panel in different environments were shown in Table 1. The *H*_*B*_^*2*^ and BLUPs were used for evaluating the genetic variance components of the target traits (S2 Table and S2 Fig). The *H*_*B*_^*2*^ of GY and related traits had a wide range from 0.43 (LAI) to 0.92 (PH). Among three major yield components, TKW (0.88) had the highest *H*_*B*_^*2*^, followed by KPS (0.75), and ESN (0.64).

**Table 1:** Phenotypic variation and broad sense heritability of the grain yield and related traits.

Trait	E^a^	Mean±SD	Range	*H*_*B*_^*2*^	Trait	E	Mean±SD	Range	*H*_*B*_^*2*^
**MSN**	E1	1337±230	833–2054	0.73	**RLW**	E1	1.9±0.1	1.7–2.2	0.85
	E2	1263±241	803–2201			E2	2.0±0.1	1.7–2.4	
	E3	1136±242	714–2187			E3	1.9±0.1	1.7–2.2	
	E4	1165±256	617–2263			E4	2.0±0.1	1.8–2.3	
**ESN**	E1	546±102	400–935	0.64	**LAI**	E1	-	-	0.43
	E2	555±90	384–851			E2	-	-	
	E3	591±116	314–924			E3	5.1±0.9	3.5–7.5	
	E4	615±128	307–951			E4	4.8±1.1	2.5–7.2	
**PES**	E1	0.41±0.07	0.29–0.64	0.56	**PH**	E1	81.6±8.5	64.0–104.5	0.92
	E2	0.45±0.08	0.31–0.66			E2	81.9±8.7	64.1–106.5	
	E3	0.53±0.10	0.30–0.87			E3	83.3±9.8	63.7–121.0	
	E4	0.54±0.11	0.31–0.92			E4	83.6±10.1	65.7–117.2	
**KPS**	E1	45.8±6.3	29.3–60.9	0.75	**GY**	E1	3.2±0.6	1.8–4.5	0.63
	E2	47.0±6.0	31.6–60.8			E2	3.0±0.7	1.2–4.4	
	E3	44.7±6.0	33.2–62.9			E3	3.6±0.5	1.0–4.5	
	E4	42.9±6.1	28.6–57.5			E4	3.5±0.5	2.0–4.6	
**TKW**	E1	43.0±3.7	33.0–56.8	0.88					
	E2	41.6±3.7	30.9–57.3						
	E3	43.8±4.2	29.4–55.5						
	E4	43.3±4.0	32.8–53.3						

^a^E1, E2, E3, and E4 represent Dayangdian (2014–2015), Guohe (2014–2015), Dayangdian (2015–2016) and Guohe (2015–2016), respectively.

MSN, maximum number of shoots per square meter; ESN, effective number of spikes per square meter; PES, percentage of effective spike; KPS, number of kernels per spike; TKW, thousand-kernel weight; RLW, the ratio of kernel length/kernel width; LAI, leaf-area index; PH, plant height; GY, grain yield

The BLUPs for GY and related traits were positively correlated with the measured data in different environments (0.61–0.97) (S3 Table). There was no significant correlation between GY and MSN, ESN, or PES. However, KPS and TKW were positively correlated with GY (0.20 and 0.34, respectively) (S4 Table). A significantly positive correlation (0.62) was identified between MSN and ESN, but no correlation between KPS and TKW. Additionally, we observed a positive correlation (0.35) between ESN and LAI. These results fully revealed the complexity and instability of wheat yield formation.

### Model-based population structure, principal component, and kinship analyses

**Fig 1:**
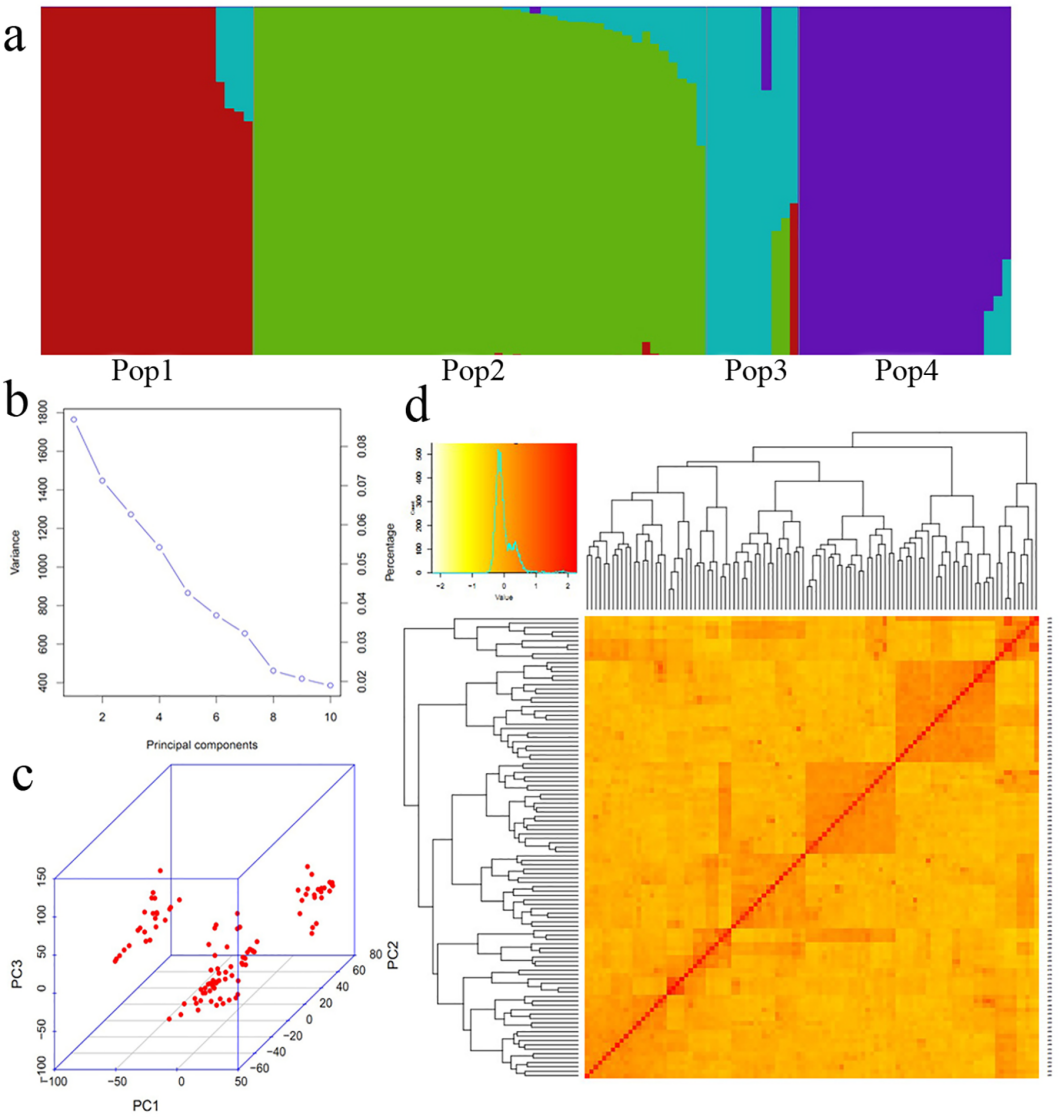
Population structure, principal component, and kinship analyses, respectively, with the district plot (a), the screen plot (b), and the genetic clustering heat map (d). The district plot (a) was generated using the mean of the variation posterior distribution over inferred admixture proportions. The screen plot (b) was generated with the changes in variances in each principal component. Three-dimensional plot of the first three principal components (c) along with the results of the kinship analysis with the genetic clustering heat map (d) was created with a kinship matrix for evaluating the genetic differences among 105 wheat varieties.

To effectively evaluate population compositions, a *Q* matrix (*K* = 4) and kinship matrix (K*) as well as a *PC* matrix (PC8) and kinship matrix (K*) were used as the covariates for a subsequent association study. The panel of 105 wheat varieties and advanced lines was divided into four subpopulations based on model complexity. The 105 elite breeding varieties were assigned to each subpopulation according to ancestry contributions (Fig 1a). A significant change in the variances was detected in the eighth PC (Fig 1b), indicating the cumulative variance contribution (> 40%) was relatively high for the first eight principal components. These varieties were assigned to three genetic clusters in a three-dimensional plot of the first three principal components (i.e., PC1, PC2, and PC3) (Fig 1c). Genetic clustering with the kinship matrix indicated that the association mapping panel was mainly divided into three groups, with considerable genetic differences among the varieties (i.e., red to yellow in the clustering heat map of Fig 1d).

### Marker–trait association analysis

According to the BIC values of different traits, a *Q* + K* model was selected for association analysis of MSN, ESN, KPS, TKW, LAI, PH, and GY. A PC8 + K* model was chosen for PES and RLW (S5 Table). In total, 24 highly significant MTAs (*P* < 0.001) were detected on chromosomes 1A, 1D, 2A (2), 3B, 4A (2), 4B, 5A (4), 5B (4), 5D, 6B (2), 7A (2), and 7B (3) using the *Q* + K* or PC8 + K* models for these traits. These MTAs could explain 10.86–20.27% of the phenotypic variance (Table 2).

**Table 2:** Details regarding the significant marker–trait associations (*P* < 0.001) for grain yield and related traits.

Trait	Marker	Chr.	Pos (cM)	Alleles	*P* (×10^−4^)					R^2^ (%)
					BLUP	E1	E2	E3	E4	BLUP
**MSN**	*RFL_Contig2531_2144*	4A	145.19	T/C	6.73	-	-	5.79	-	12.37
	*Kukri_c49033_52*	5A	120.44	C/T	8.76	-	-	-	1.73	11.33
**ESN**	*Ku_c16809_845*	1D	78.36	G/A	6.53	-	5.36	-	-	12.18
	*wsnp_Ex_c15944_24350833*	3B	62.57	G/A	7.88	-	-	-	-	15.47
	*Excalibur_c15944_70*	3B	62.67	A/G	8.89	-	1.30	-	-	15.2
	*Kukri_c12563_52*	4A	66.28	C/T	5.72	-	-	-	4.24	15.82
	*Kukri_rep_c104277_1326*	4B	26.00	A/G	5.99	-	-	4.55	-	16.01
	*Excalibur_c55463_232*	4B	26.00	T/C	8.06	-	-	9.79	-	15.3
**PES**	*Kukri_c46939_83*	1A	30.99	C/T	8.34	-	9.93	-	-	14.95
	*Excalibur_c33675_410*	5B	94.89	A/G	8.06	-	-	-	-	14.93
**KPS**	*Kukri_c14516_224*	7A	130.27	C/T	6.72	-	7.48	-	5.51	12.45
	*Tdurum_contig10002_533*	7A	130.27	C/T	8.09	-	8.89	-	6.41	12.21
	*BS00108184_51*	7A	130.27	A/C	9.56	-	9.43	-	7.89	11.78
**TKW**	*BS00073670_51*	5A	84.13	A/G	3.88	5.27	9.19	-	-	12.9
	*wsnp_Ex_c1138_2185522*	5A	86.36	A/G	4.01	7.10	7.24	-	-	12.83
	*Tdurum_contig71499_211*	5A	86.36	A/G	7.60	-	-	-	-	12.13
	***Excalibur_c14451_1313***	7A	156.23	G/A	2.48	2.53	4.25	-	0.52	13.95
	***Kukri_c19251_579***	7A	156.23	C/T	2.53	2.72	4.02	-	0.52	13.86
**RLW**	*wsnp_Ex_c2526_4715978*	5A	99.56	C/A	9.33	-	-	-	8.48	14.1
	*Ex_c24031_300*	5B	212.43	G/T	7.20	-	-	5.23	1.21	11.42
	***Tdurum_contig14046_364***	6B	67.24	C/T	0.33	2.60	8.22	2.25	1.35	17.68
	*wsnp_Ex_c24376_33618864*	7B	52.18	C/T	9.26	-	-	5.30	-	10.86
	*wsnp_Ex_c24376_33619527*	7B	52.18	T/G	9.26	-	-	5.30	-	10.86
**LAI**	*GENE-1177_195*	2A	62.51	G/T	5.18	-	-	-	-	12.62
**PH**	*Excalibur_c1925_2569*	5B	131.79	T/C	9.01	-	-	-	-	14.74
	*Kukri_c9285_762*	5D	200.74	G/A	3.65	-	-	7.27	0.18	13.74
	*Kukri_rep_c106092_300*	6B	113.67	C/T	5.38	-	-	0.38	1.15	16.33
	***wsnp_Ex_c11003_17857272***	7B	77.13	A/G	3.03	4.80	9.55	-	1.57	17.72
	***wsnp_Ex_rep_c68762_67626384***	7B	129.77	G/A	1.13	3.92	-	0.34	0.30	16.32
	***Excalibur_c50612_409***	7B	129.77	A/G	1.54	-	9.24	2.10	0.33	20.27
	*Tdurum_contig77073_193*	7B	129.77	T/C	2.04	-	-	2.51	0.39	19.94
**GY**	*Ex_c6937_1992*	2A	156.09	A/G	5.12	-	-	-	-	12.5
	*RAC875_c2926_371*	5A	32.33	G/A	9.65	-	-	-	-	14.5
	*wsnp_Ku_c7890_13513783*	5A	32.33	C/T	9.74	-	-	-	-	14.47
	*wsnp_BQ166999B_Ta_2_1*	5B	39.64	T/G	7.29	-	-	-	-	15.91
	*Excalibur_c92223_97*	5B	39.64	G/A	8.40	-	-	-	-	11.5
	*Tdurum_contig52439_196*	5B	40.56	C/T	3.88	-	-	-	-	15.74
	*RAC875_c31299_1215*	6B	110.45	T/C	3.88	-	-	-	-	12.38

Note: MSN, maximum number of shoots per square meter; ESN, effective number of spikes per square meter; PES, percentage of effective spikes; KPS, number of kernels per spike; TKW, thousand-kernel weight; RLW, the ratio of kernel length/kernel width; LAI, leaf-area index; PH, plant height; GY, grain yield per plot. E1, E2, E3, and E4 represent Dayangdian (2014–2015), Guohe (2014–2015), Dayangdian (2015–2016) and Guohe (2015–2016), respectively. Markers highlighted in bold were detected in more than three environments (*P* < 0.001), and markers underlined with the genetic distance less than 5 cM was assumed as one MTA.

Four MTAs for ESN were identified on chromosomes 1D (*Ku_c16809_845*, 78.36 cM), 3B (*wsnp_Ex_c15944_24350833*, 62.57 cM; *Excalibur_c15944_70*, 62.67 cM), 4A (*Kukri_c12563_52*, 66.28 cM), and 4B (*Kukri_rep_c104277_1326* and *Excalibur_c55463_232*, 26.00 cM), explaining 12.18–16.01% of the phenotypic variance. However, all SNPs related to ESN were only detected in one environment or not (*P* < 0.001), suggesting the genetic instability of these MTAs in different environments.

For KPS, we detected only one MTA (*Kukri_c14516_224*, *Tdurum_contig10002_533*, and *BS00108184_51*) on chromosome 7A (130.27 cM), explaining 11.78–12.45% of the phenotypic variance.

For TKW, one MTA harbored three SNP markers (*BS00073670_51*, *wsnp_Ex_c1138_2185522*, and *Tdurum_contig71499_211*) on chromosome 5A (84.13–86.36 cM), accounting for an average phenotypic variation of 12.62%. Another one with two SNPs (*Excalibur_c14451_1313* and *Kukri_c19251_579*) significantly associated in three environments (E1, E2, and E4) (*P* < 0.001) on chromosome 7A (156.23 cM), explained 13.91% of the TKW variation, which implied this MTA is a major one.

Four MTAs were identified for RLW on chromosomes 5A (*wsnp_Ex_c2526_4715978*, 99.56 cM), 5B (*Ex_c24031_300*, 212.43 cM), 6B (*Tdurum_contig14046_364*, 67.24 cM), and 7B (*wsnp_Ex_c24376_33618864* and *wsnp_Ex_c24376_33619527*, 52.18 cM). Of them, the MTA on 6B was more stable and significant (*P* < 0.001) in four environments (E1, E2, E3, and E4), explaining a higher phenotypic variance (17.68%), which implied this region covered a credible QTL.

Five MTAs for PH on chromosomes 5B (*Excalibur_c1925_2569*, 131.79 cM), 5D (*Kukri_c9285_762*, 200.74 cM), 6B (*Kukri_rep_c106092_300*, 113.67 cM), and 7B (*wsnp_Ex_c11003_17857272*, 77.13 cM; *wsnp_Ex_rep_c68762_67626384*, *Excalibur_c50612_409*, and *Tdurum_contig77073_193*, 129.77 cM) explained 13.74–20.27% of the phenotypic variance. Of them, both two MTAs on 7B (77.13 cM and 129.77 cM) were identified in three environments, indicating two independent major loci on 7B. Of these associated SNPs, *wsnp_Ex_rep_c68762_67626384* on 7B was more significantly associated with PH, suggesting the importance of this region.

Four MTAs associated with GY were identified on chromosomes 2A, 5A, 5B, and 6B, respectively, with phenotypic contributions ranging from 11.50% (*Excalibur_c92223_97*) to 15.91% (*wsnp_BQ166999B_Ta_2_1*), and an average of 13.86%. Similar to ESN, all MTAs for GY showed a poor genetic stability in different environments.

### CAPS marker development and validation of a SNP for TKW on chromosome 5A

**Fig 2:**
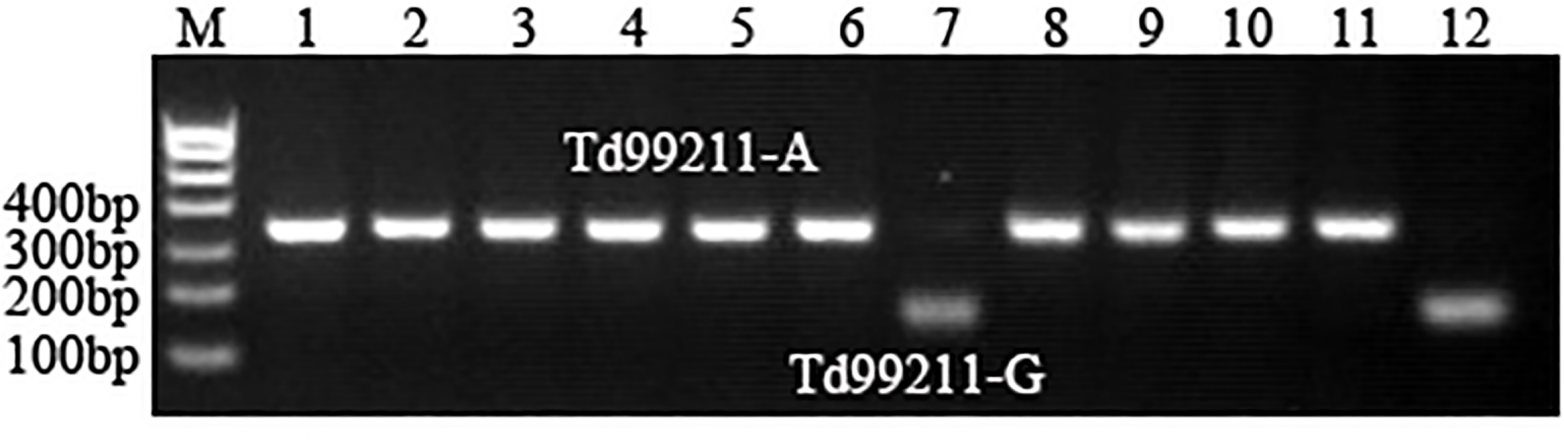
Two allelic variations (*Td99211-A* and *Td99211-G*) of the CAPS marker (*Td99211*) digested by *AluI* in part wheat materials.

A SNP (*Tdurum_contig71499_211*) on chromosome 5A for TKW was developed into the CAPS marker (*Td99211*), and genotyped in 372 wheat varieties (lines) and 188 lines from the RIL population of Yangxiaomai × Zhongyou 9507 cross. Two allelic variations were detected, designated *Td99211-A* and *Td99211-G* (Fig 2). There was a significant difference in TKW between the two alleles in the two populations (*P* < 0.01), and the varieties (lines) harboring *Td99211-G* had a higher TKW compared with those carrying *Td99211-A* (Table 3).

**Table 3:** Validation of a SNP (*Tdurum_contig71499_211*) for TKW on chromosome 5A in the natural population (NP) composed of 372 wheat varieties (lines) and the RIL population derived from the cross of Yangxiaomai × Zhongyou 9507 across environments.

Population	genotype	Number	TKW (2015–2016)			TKW (2016–2017)		
			Mean ± SD	*t*-test	R^2^ (%)	Mean ± SD	*t*-test	R^2^ (%)
**NP (n = 372)**	*Td99211-A*	323	32.50 ± 8.32	3.606**	3.4	32.39 ± 8.15	3.670**	3.5
	*Td99211-G*	49	37.11 ± 8.36			36.97 ± 8.10		
**RILs (n = 188)**	*Td99211-A*	96	33.34 ± 4.42	6.572**	18.8	33.49 ± 3.37	4.606**	10.2
	*Td99211-G*	92	37.68 ± 4.86			35.75 ± 3.35		

Note: TKW, thousand-kernel weight;

**, significant at 0.01 probability level.

## Discussion

### Analysis of phenotype heritability

In the current study, 105 elite wheat varieties and advanced lines had extremely diverse genetic backgrounds and highly variable phenotypes. Because of the excellent agronomic traits, most of them were widely used as parents in breeding [22]. However, the complex genetic relationships between GY and related traits dramatically hindered relevant research process of GY formation and the breeding application of obtained achievements. Therefore, investigations of MTAs for GY and related traits in these varieties will provide useful information for wheat breeding programs.

The yield-related traits belong to typical quantitative traits controlled by multiple QTLs, and highly vulnerable to environmental factors. The use of BLUPs can eliminate the environmental deviation and estimate the real individual breeding value [23–25]. In the present study, we investigated nine yield-related traits, including MSN, ESN, PES, KPS, TKW, LAI, RLW, PH, and GY, and analyzed their BLUP values. There was a highly significant correlation between BLUP values and the measured values in different environments, indicating that the BLUP values are suitable for GWAS.

In addition, we also analyzed the *H*_*B*_^*2*^ values of the above nine traits. The PH had highest *H*_*B*_^*2*^ values (0.92), followed by TKW (0.88) and RLW (0.85), while the LAI was the lowest (0.43). The difference of heritability is consistent with GWAS results, that is, only one SNP for LAI was detected, while four major MTAs for PH (2), TKW (1), and RLW (1) were stably identified across environments.

### Evaluation of population structure

Because of the limitations of the Structure [39] and Admixture [40] programs regarding the number of markers used for population structure analysis, only a small proportion of SNP markers were utilized in previous studies [13–18]. This probably produces false-positive results. In contrast, we used 31,250 effective SNP markers to accurately analyze the population structure (Fig 1a) with the fastStructure algorithm that estimates the approximate posterior distributions on ancestry proportions in two orders of magnitude faster than Structure, with ancestry estimates and prediction accuracies comparable to those of Admixture [30]. The considerable improvement in runtime and comparable accuracies of fastStructure enables the application of this algorithm for analyzing large genotype data sets, generating results clearly different from those of previous studies.

The use of different models is suitable for studying different traits, but blindly using the *Q* + K* model for all traits probably results in an over-correction of the population structure and some false-negative results [17, 32, 36]. Therefore, the principal component (Fig 1b and 1c) and kinship (Fig 1d) of the association mapping panel were also accurately calculated with all 31,250 effective SNP markers to build the *Q* + K* and PC8 + K* models.

### Comparison of the present study with previous researches

Based on 90K-derived genetic map described by Wang et al. [29] (S6 Table), we further compare the partial MTAs identified in the present study with previous researches. In this study, the MTA (*Ex_c24031_300*) significantly associated with RLW was detected on chromosome 5B (212.43 cM). Chen et al. [20] also identified a MTA (*IACX2594*) for RLW in the same genetic position (212.43 cM) using a high-density Illumina iSelect 90K single nucleotide polymorphism assay in a Chinese winter wheat population. For GY, we identified a MTA (*RAC875_c2926_371* and *wsnp_Ku_c7890_13513783*) on chromosome 5A, which was only 3.62 cM from the QTL (*wsnp_Ex_c31830_40573624* and *wsnp_Ex_rep_c69526_68472665*) for GY reported by Li et al. [8]. Therefore, we suggest that the above two QTLs belong to the same locus controlling the GY trait. In addition, in the present study, we also identified a major MTA (*Excalibur_c14451_1313* and *Kukri_c19251_579*) for TKW on chromosome 7A (156.23 cM), which was adjacent to the QTLs (*wsnp_Ex_c11047_17915103*, *wsnp_Ku_c8437_14341371*, *BS00021657_51*, *wsnp_JD_c20555_18262317*, and *CAP7_c2350_105*) controlling TKW reported by Li et al. [8] and Su et al. [10]. For PH, a significant MTA (*Kukri_c9285_762*) was detected on chromosome 5D in this study, which was close to *BS00089597_51* (known as *GA20ox1* in rice) associated with PH reported by Zanke et al. [15]. In the present study, the SNPs within 5 cM associated with the same traits were assumed as one MTA/QTL. Therefore, the MTAs/QTLs for TKW (7A) and PH (5D) identified in the present study were the same loci with those previously reported by Zanke et al. [15], Li et al. [8] and Su et al. [10]. Notably, no MTA or QTL associated with PH was reported on chromosome 7B, suggesting that the two MTAs on 7B (77.13 and 129.77 cM) identified in the present study are likely to be novel.

### MTAs with pleiotropic effects

We detected a MTA on chromosome 6B with PH (*Kukri_rep_c106092_300*, 113.67 cM) and GY (*RAC875_c31299_1215*, 110.45 cM), indicating the importance of PH to GY. However, several MTAs for different traits were detected in the same or neighboring positions as those identified in previous studies. For example, *wsnp_Ex_c24376_33618864* for RLW on chromosome 7B (52.18 cM) was also identified for PH by Zanke et al. [15]. *Wsnp_Ex_c11003_17857272* for PH on chromosome 7B (77.13 cM) was detected for TKW (*Ex_c12057_797*, 77.13cM) [18]. *Wsnp_Ex_c15944_24350833* and *Excalibur_c15944_70* for ESN on chromosome 2A were located in the same position (62.57 cM) as *Kukri_c21467_571* for TKW [18] and *wsnp_JD_c8158_9193784* for KW [20]. Additionally, the MTA was also close to *Kukri_c48750_1372* (61.89 cM) for chlorophyll content (measured as SPAD value) during grain filling [17] and BS00074688_51 (65.55 cM) for days-to-heading [19]. *Tdurum_contig14046_364* for RLW on chromosome 6B was only 0.84 cM from the QTLs for the number of spikes per square meter (*wsnp_Ra_c14498_667649*, *wsnp_Ex_c34011_42398664*, and *wsnp_Ex_rep_c67012_65465394*) [8]. *RAC875_c31299_1215* for GY on chromosome 6B was located only 0.59 cM from the QTLs for TKW (*wsnp_Ex_c3025_5587183*, *wsnp_Ex_rep_c66342_64519823*, *wsnp_Ex_rep_c69373_68311942*, and *wsnp_Ex_rep_c69373_68312188*) [8], and was also close to the MTA (*RAC875_rep_c71463_98*) for PH [15]. These results revealed the pleiotropism of QTLs/MTAs for the GY and related traits, which may be due to the complex relationships among these traits.

### Development of CAPS marker for TKW on chromosome 5A and its application in wheat breeding

The SNP (*Tdurum_contig71499_211*) on chromosome 5A was identified to be significantly associated with TKW based on BLUP values, and a CAPS marker (*Td99211*) for the SNP was successfully developed. Using 372 wheat varieties (lines) and 188 lines from the RIL population of Yangxiaomai × Zhongyou 9507 cross, we further validated the association of the CAPS marker with TKW. Moreover, the *Td99211-G* allele was associated with higher TKW compared with *Td99211-A*, and thus considered as a favorable allele. Notably, only 49 (13.17%) harbored the *Td99211-G* allele in 372 wheat varieties (lines), indicating the *Td99211-G* allele have not been widely utilized in genetic improvement of wheat yield. By contrast, the favorable variations of several genes controlling TKW, such as *TaGW2* [41], *TaCWI* [42], *TaGS-D1* [43], *TaGASR7-A1* [44], have been positively selected in wheat breeding. However, the candidate genes controlling TKW on chromosome 5A had not been reported in previous studies. Therefore, cloning of the target gene controlling TKW on chromosome 5A is necessary for pyramiding breeding for wheat yield.

## Supporting information

S1 Table Distribution of 31,250 and 15,430 effective single nucleotide polymorphism markers throughout the wheat genome. (XLSX)

S2 Table The phenotypic BLUPs of different varieties. (XLSX)

S3 Table Correlations between best linear unbiased predictions and the measured values among different environments. (XLSX)

S4 Table Correlations among best linear unbiased predictions of the grain yield and related traits. (XLSX)

S5 Table Bayesian information criterion values for various traits analyzed using a model with Q + K* or PC8 + K* covariates. (XLSX)

S6 Table The comparison of 90K markers identified in different studies. (XLSX)

S1 Fig The GWAS panel of 105 winter wheat varieties with abundant phenotypic variation used in this study. (TIF)

S2 Fig Frequency distributions of different phenotypic BLUPs. (TIF)
