# Supplementary figures and images for: Genome-wide association study for grain yield and related traits in elite wheat varieties and advanced lines using SNP markers
Source: PLoS One. 2017 Nov 27;12(11):e0188662. doi: 10.1371/journal.pone.0188662 (PMC5703539; doi:10.1371/journal.pone.0188662)

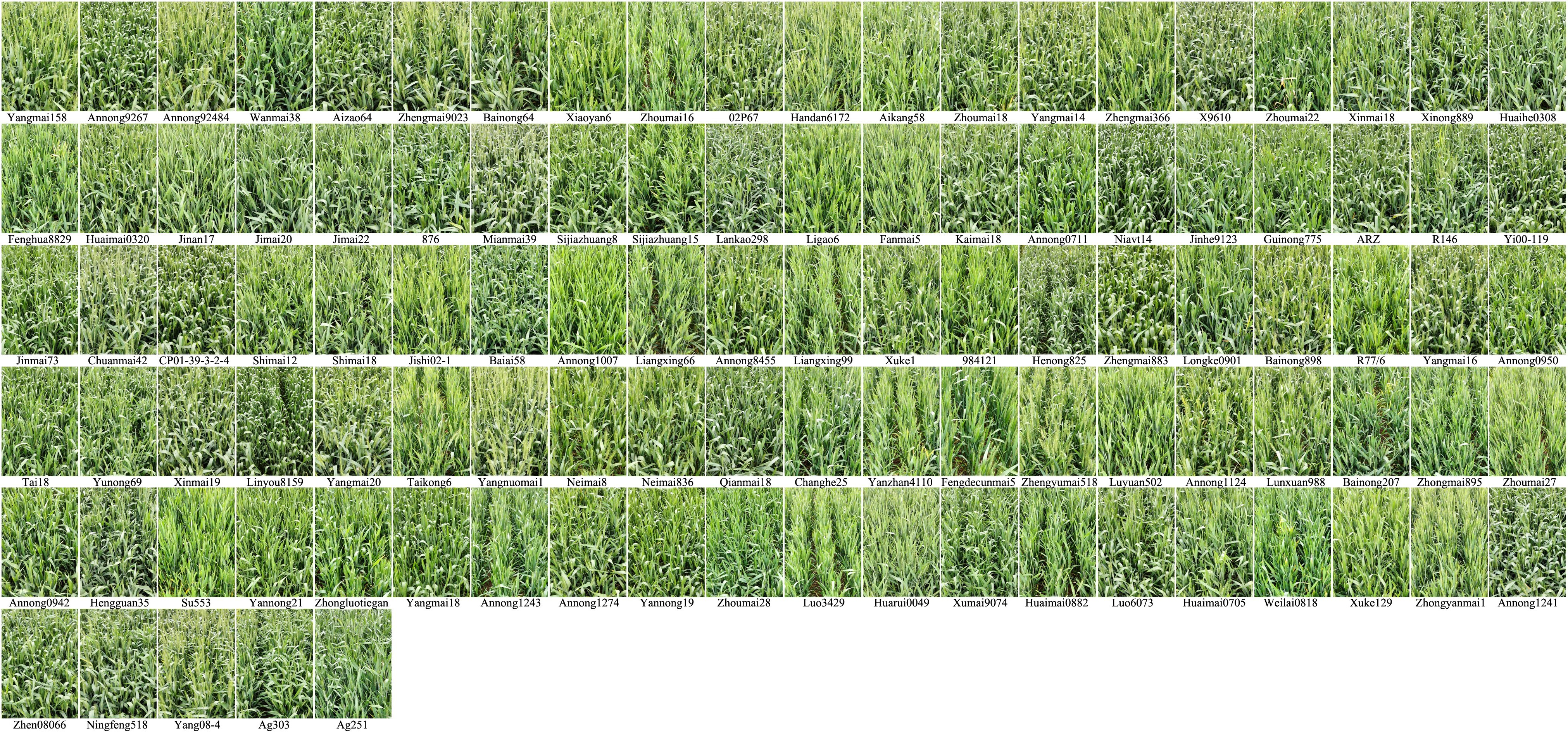

Supplement: S1 Fig — (TIF) [file pone.0188662.s007.tif]

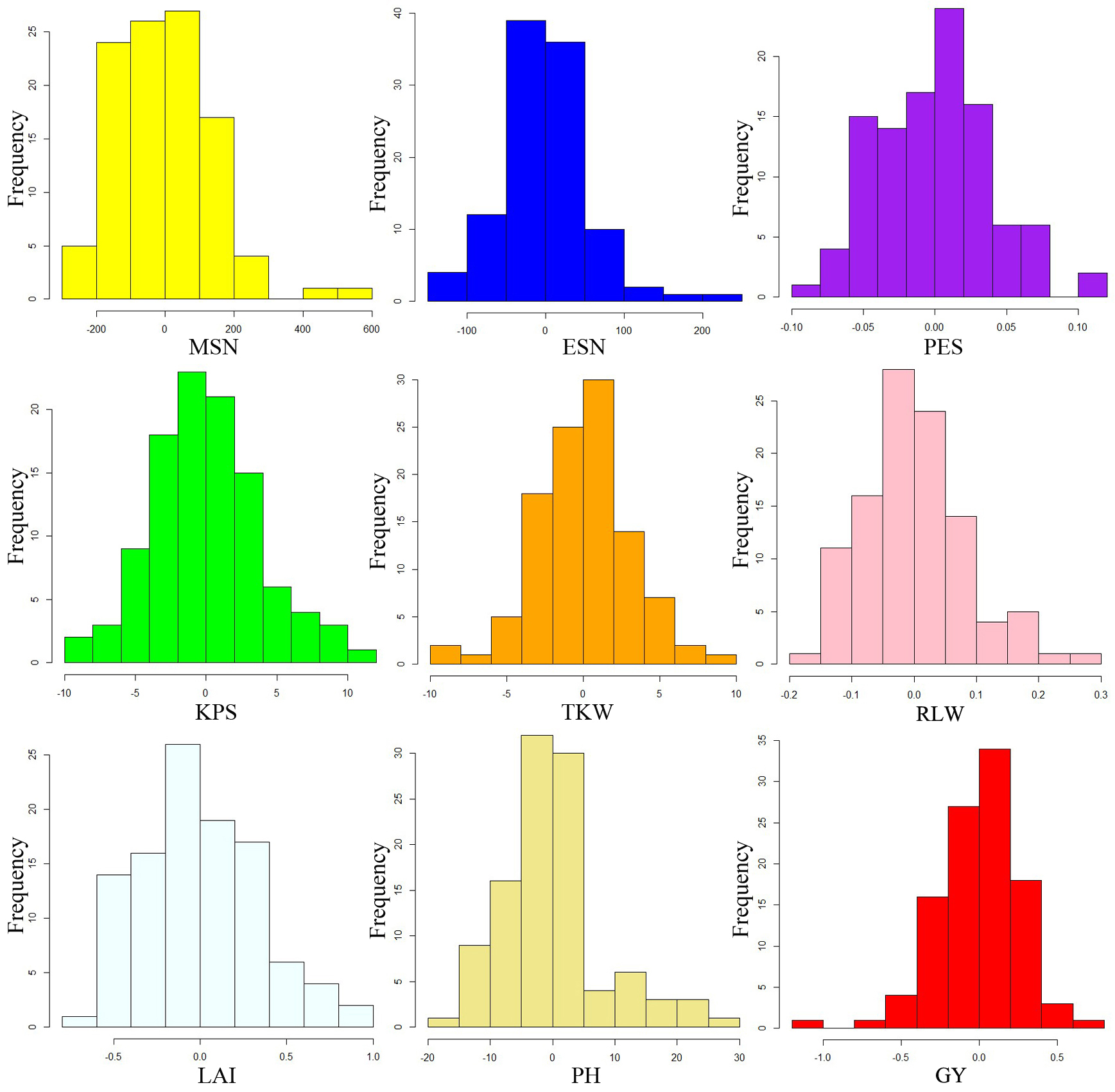

Supplement: S2 Fig — (TIF) [file pone.0188662.s008.tif]
